# Preparation and Physicochemical Properties of Modified Corn Starch–Chitosan Biodegradable Films

**DOI:** 10.3390/polym13244431

**Published:** 2021-12-17

**Authors:** Enrique Javier Jiménez-Regalado, Carolina Caicedo, Abril Fonseca-García, Claudia Cecilia Rivera-Vallejo, Rocio Yaneli Aguirre-Loredo

**Affiliations:** 1 Centro de Investigación en Química Aplicada (CIQA), Blvd. Enrique Reyna Hermosillo 140, Saltillo, Coahuila 25294, Mexico; enrique.jimenez@ciqa.edu.mx (E.J.J.-R.); abril.fonseca@ciqa.edu.mx (A.F.-G.); claudia.rivera@ciqa.edu.mx (C.C.R.-V.); 2 Grupo de Investigación en Química y Biotecnología (QUIBIO), Facultad de Ciencias Básicas, Universidad Santiago de Cali, Pampalinda, Santiago de Cali 760035, Colombia; carolina.caicedo03@usc.edu.co; 3 Consejo Nacional de Ciencia y Tecnología (CONACYT)—CIQA, Blvd. Enrique Reyna Hermosillo 140, Saltillo, Coahuila 25294, Mexico

**Keywords:** biodegradable film, thermoplastic starch, chitosan, mechanical properties, water-vapor permeability

## Abstract

Starch is a biopolymer with enormous potential for generating new biodegradable packages due to its easy availability and low cost. However, due to its weak functional properties, limitation of its interaction with some hydroxyl groups and evaluation of blends with other polymers are necessary in order to improve its performance. Glycerol-plasticized acetylated corn starch films were developed using the casting method, and the impact of incorporating chitosan (TPS:CH) in various proportions (75:25, 50:50, and 25:75 *v*/*v*) was studied in the present research. The effect of chitosan ratios on the physical, mechanical, water-vapor barrier, and thermal properties of the film was studied. Chitosan-protonated amino groups promoted the formation of intermolecular bonds, improving tensile strength, thermal stability, hydrophobicity, water adsorption capacity, and the gas barrier of starch films. The results show that the film composed of TPS25-CH75 proved to be the best barrier to water vapor; thus, these composite films are excellent choices for developing biodegradable packaging for the food industry.

## 1. Introduction

The world is well aware that humanity is facing a critical pollution problem. Plastic, particularly as used in the food industry for packaging, conservation, transport, and consumption, is one of the most commonly discarded materials. Thus, several research propositions have been made to develop more environmentally friendly materials to replace conventional synthetic plastics. These new materials can be obtained from various mixtures of biodegradable polymers with additives such as plasticizers, antioxidants, and antimicrobials.

Starch, a natural polymer, is an excellent choice for making low-cost biodegradable packaging materials due to its affordability and wide availability. Starch is composed of two molecules: amylose and amylopectin. Amylose is a linear polysaccharide comprising α-1,4-linked D-glucopyranose, while amylopectin is a highly branched molecule consisting of chains of α-D-glucopyranosyl residues connected by 1,4 and 1,6 bonds. However, starch-based materials are brittle and susceptible to moisture; thus, several methods have been developed to improve their functional performances. Starch can be modified in various ways to change its processing properties, as well as the materials generated. One method of modification is through chemical processes such as esterification and acetylation. Acetyl groups break the inter- and intramolecular bonds of the starch, weakening its structure and allowing a higher mobility of the polymeric chains of the amorphous region [1,2]. Acetylated starch has a lower gelatinization temperature than regular starch, along with a slower retrograde rate [1]. Films obtained with modified starch show higher elongation and lower water-vapor permeability than films based on native starch [3]. Biodegradable starch films usually have good mechanical strength; however, they have a high permeability to gases, such as water vapor. Another way to modify the functional properties of starch-based materials is to blend them with other polymers or additives. Starch is mixed with other biopolymers, such as polyvinyl alcohol and chitosan, to improve its physicochemical and functional properties [1,4,5].

Chitosan is a natural linear polysaccharide derived from chitin extracted from the exoskeleton of crustaceans, a byproduct of waste from the fishing industry [2]. It is biodegradable, non-toxic, biofunctional, biocompatible, and shows antimicrobial and antifungal activity due to its cationic nature [6]. Researchers have shown that mixtures of starches with chitosan can form films the mechanical and barrier performance of which highly depends on the concentration of chitosan added to the starch and its deacetylation degree [7,8].

The objective of this study was to evaluate how acetylated corn starch-chitosan (TPS:CH) affected the mechanical, gas barrier, thermal, and water-vapor-sorption properties of a biodegradable film based on modified (acetylated) starch films plasticized with glycerol in three different ratios (75:25, 50:50, and 25:75 *v*/*v*).

## 2. Materials and Methods

### 2.1. Materials

Modified food starch (Pure-Gel, B994) obtained from Grain Processing Corporation (Muscatine, IA, USA) and chitosan (90% deacetylation degree) from Alfadelta Materias Primas (Naucalpan, Mexico) were used as biodegradable polymers. Anhydrous glycerol from J.T. Baker (Ciudad de Mexico, Mexico) was used as a plasticizer. Productos Quimicos Monterrey (Monterrey, Mexico) provided glacial acetic acid. Lithium chloride (LiCl), potassium acetate (CH_3_COOK), magnesium chloride (MgCl_2_), potassium carbonate (K_2_CO_3_), sodium bromide (NaBr), sodium chloride (NaCl), potassium chloride (KCl), and barium chloride (BaCl_2_) (Jalmek Cientifica, Monterrey, Mexico) were used to prepare supersaturated saline solutions to obtain environments with an equilibrium relative humidity (RH) of 11%, 22%, 32%, 43%, 57%, 75%, 84%, and 90%, respectively.

### 2.2. Preparation of Films

To prepare the film-forming solutions, five starch:chitosan (TPS:CH) ratios were developed: 0:100, 75:25, 50:50, 25:75, and 0:00. They were denoted as TPS100-CH0, TPS75-CH25, TPS50-CH50, TPS25-CH75, and TPS0-CH100, respectively. The corresponding amount of starch (for preparation at 6% *w*/*v*) was dispersed in distilled water, and the plasticizer was added at a concentration of 30% (*w*/*w* of total polymers). When the starch dispersion reached 50 °C, a corresponding volume (mL) of a 1% (*w*/*v*) solution of chitosan (dissolved in 1% *v*/*v* acetic-acid solution) was added. The filmogenic solutions were constantly stirred, and the temperature was increased to 90–92 °C for 7 min to allow the starch to gelatinize. Then, 100 mL of the filmogenic solution was poured into 150 × 150 mm acrylic molds and dried at 65 °C for 5 h in an oven (OV-470A model, Thermolyne, Blue M, Blue Island, IL, USA). Before evaluating the functional properties, the dried films were peeled and stored in desiccators containing a supersaturated solution of NaBr for 48 h at room temperature (~25 °C) and 54% RH.

### 2.3. Thickness

The film thickness was measured in 10 random positions across the entire surface of the material using a digital micrometer (Mitutoyo, model C112EXB, Aurora, IL, USA, precision 0.001 mm). The average and standard deviation of 10 film samples were obtained.

### 2.4. Water Solubility

The capacity and resistance to being in contact with the liquid water of the materials were determined with the solubilization capacity in water at room temperature (25 °C), following the Fonseca-García and Jiménez-Regalado [9] methodology.

### 2.5. Contact Angle

To determine the hydrophilicity of the films, the contact angle formed by a 3 µL drop of distilled water on the opaque side of the films (mold contact) was measured. To perform this analysis, we used an instrument from Rame-Hart Co. (Succasunna, NJ, USA) coupled with a camera; a video of the raindrop formed on the films was recorded. The angle formed in the water drop was analyzed 20 s after it had been in contact with the films. The contact angle was measured in triplicate for each film.

### 2.6. Fourier Transform Infrared (FT-IR) Analysis

The chemical structure of films was analyzed by Fourier transform infrared (FT-IR) using a NICOLET iS10 spectrometer of Thermo Fisher SCIENTIFIC (Madison, WI, USA). The measurements were performed with attenuated total reflection (ATR).

### 2.7. X-ray Diffraction (XRD) Analysis

The arrangement of the structural matrix of the films was determined using a Siemens D500 powder diffractometer, following the methodology proposed by Fonseca-García and Jiménez-Regalado [9].

### 2.8. Thermal Properties

The thermal behavior of the materials was determined using differential scanning calorimetry (DSC) and thermogravimetric analysis (TGA) techniques with DSC 2500 Discovery (TA Instruments, New Castle, DE, USA) and TGA Q500 (TA Instruments, New Castle, DE, USA) analyzer equipment, as well as the methodology proposed by Fonseca-García and Jiménez-Regalado [9].

### 2.9. Water-Vapor Permeability (WVP)

Water-vapor permeability was determined according to the methodology proposed by Aguirre-Loredo and Rodríguez-Hernández [10] following the ASTM E96-02 [11] standard. The permeability cell comprised a glass container with an internal diameter of 24.64 mm and silica gel (~0% RH) in its interior. Film discs were mounted on a permeation cell, and the covered cell was placed in a desiccator containing a supersaturated saline solution of BaCl_2_ (90% RH), generating a water-vapor differential pressure of 2854.23 Pa. The cell was weighed seven times at 60 min intervals. The determinations were made in triplicate.

### 2.10. Adsorption Isotherms

The water-vapor adsorption isotherm of each material was determined using the static microclimate method, following the methodology developed by the COST90bis Project [12] and by Aguirre-Loredo and Rodríguez-Hernández [10], with some modifications, to determine the resistance of materials to the humidity present in the environment and to evaluate how much water they can absorb. The film samples (4 cm^2^) were placed in airtight plastic containers with supersaturated saline solutions, generating 11–90% RH. The containers were kept at room temperature (25 °C) for 14 days. The Guggenheim–Anderson–de Boer (GAB) mathematical model (Equation (1)) was used to describe the sorption isotherms of the films with different starch-chitosan ratios.

(1) $$X=\frac{X_{m}CKa_{w}}{\left(1-Ka_{w}\right)\left(1-Ka_{w}+CKa_{w}\right)}$$

where *X* is the total moisture content in the material; *a_w_* is water activity; the GAB model parameters, *Xm*, are the moisture content in the monolayer; *C* is the constant related to the sorption in the first layer; and *K* is a constant related to the sorption of water molecules in the multilayer.

### 2.11. Mechanical Properties

The mechanical properties of 10 film samples with dimensions of 10 × 50 mm were measured using a TA.XT Express Enhanced texture analyzer (Stable Micro Systems, Godalming, UK) equipped with tension grips (A/TG) at room temperature (25 °C), operating at a cross-head speed of 1 mm·s^−1^, with an initial separation of 25 mm, according to the D882-12 standard [13]. Tensile strength (TS) and the percentage of elongation at break (%E) were calculated.

### 2.12. Statistical Analysis

The results were analyzed for statistical significance by analysis of variance and Tukey’s test, with a *p* < 0.05 significance level, using the OriginPro 8.5.0 SR1 software (OriginLab Corporation, Northampton, MA, USA).

## 3. Results and Discussion

### 3.1. Thickness

A significant difference in the thickness of the polymeric materials (Table 1) as a function of the proportion of biopolymers was observed; however, this result is to be expected because the initial concentration of each of the original polymeric solutions is different, keeping in mind that starch is found at a concentration of 5%, while chitosan concentration is at 1%. However, this behavior of thickness as a function of mass was unexpected. The formulations that followed the expected behavior contained pure chitosan (TPS0-CH100, smallest mass), which presented the smallest thickness, as well as formulation based on starch-chitosan in a TPS75-CH25 ratio (higher mass of biopolymers).

### 3.2. Water Solubility and Contact Angle

One of the most important factors considered when designing and using a completely biodegradable material is the susceptibility or damage of the material due to contact with water, as most biopolymers are hydrophilic. The biodegradable films developed in this study showed increased solubilization capacity in liquid water, as observed by the values shown in Table 1. The solubility in water of pure starch films is similar to that reported by Colussi and Pinto [3] in acetylated starch and is higher than native corn starch [9]. This higher solubility of acetylated films compared to those made from native starch is due to the ease of the incursion of water molecules into the structural matrix of the modified starch film as a consequence of lower retrogradation. The solubility of the composite materials increased significantly with an increase in the proportion of chitosan present in the composite films. The thickness of the material can also influence its solubility. Although the material may have a hydrophilic nature, with increased thickness, the resistance to dissolution will be slightly higher. The data in Table 1 show that solubility was higher in materials with the least thickness and with the highest chitosan ratio.

The hydrophobic behavior shown by acetylated starch films is congruent with the fact that this biopolymer was modified by substituting hydroxyl groups with acetyl groups that promote a relative hydrophobicity such as is shown in the pristine acetylated starch film with a contact angle of 90.58°. This angle corresponds to a hydrophobic material [14].

Nevertheless, this behavior is apparently reduced in the composite films due to the addition of chitosan on the starch films. Even though in film TPS25-CH75, hydrophilicity was lower than in pristine chitosan film, this effect can be associated with polymeric chains that were ordered along the film, allowing hydroxyl groups of chitosan and starch to be on the surface of the film.

### 3.3. Fourier Transform Infrared (FT-IR) Analysis

**Figure 1:**
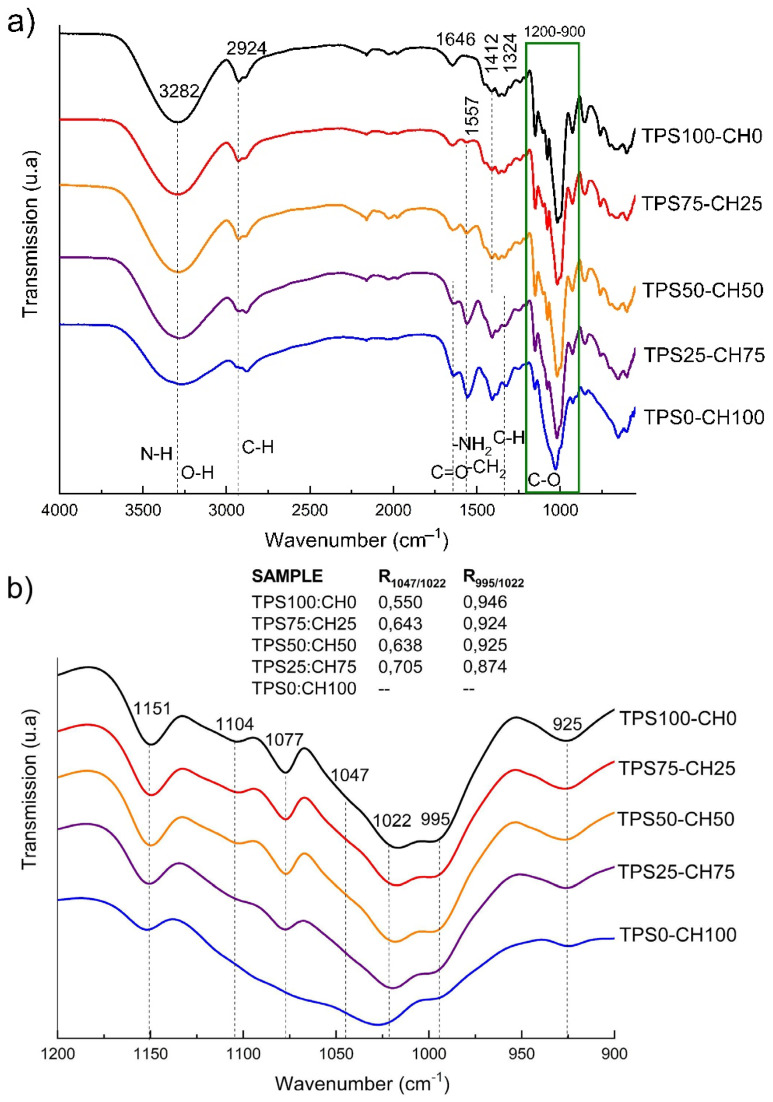
(**a**) Attenuated total reflection–Fourier transform infrared (ATR–FT-IR) spectra of starch-chitosan films. (**b**) Amplified region between 1200 cm^−1^ an 900 cm^−1^; this relates the values of R_1047/1022_ and R_995/1022_ of starch-chitosan films.

**Figure 2:**
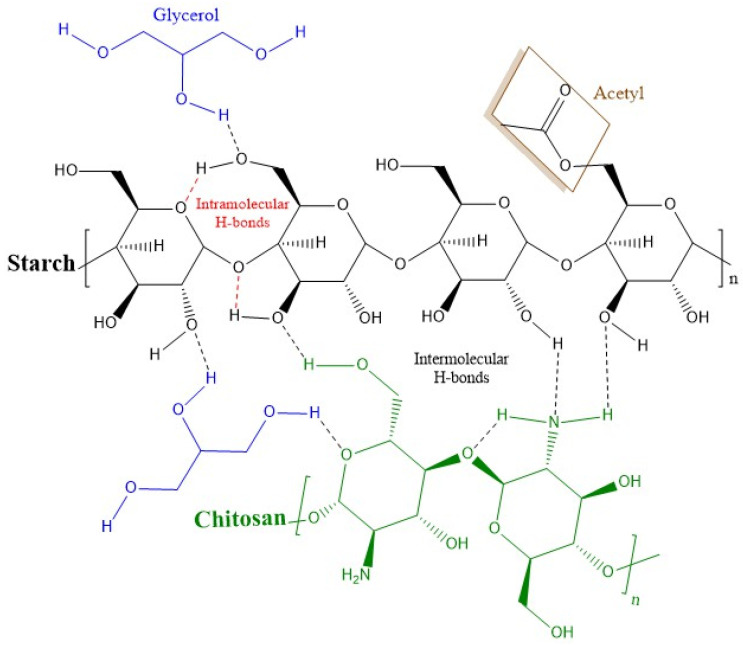
Proposed molecular interactions between starch, chitosan, and glycerol.

Figure 1 shows the spectra of blends of biodegradable films. The acetylated TPS showed characteristic bands as follows (Figure 1a). At 3282 cm^−1^, the stretching of O–H, which decreases its absorption chitosan concentration, increases. This absorption band is strong and broad, and it overlaps with –NH stretching vibration and intermolecular hydrogen bonds in the mixtures (TPS75-CH25, TPS50-CH50, and TPS25-CH75). At wavenumbers of 2924 and 2887 cm^−1^, symmetric and asymmetric stretching vibrations of methylene groups (CH_2_) are observed, respectively, in the spectrum of thermoplastic starch [15]. At 1646 cm^−1^, the bending vibration of OH (water) and carbonyl appears in chitosan at the same wavenumber. CH and CH_2_ deformations are evident at 1412 cm^−1^. In the region of 1242 and 1240 cm^−1^, O–H vibrations (bending) and C–O stretching of acetate are present. Some bands are also observed that could be attributed to the formation of hydrophilic colloids in the composite films at 1077 cm^−1^, 1018 cm^−1^, and 927 cm^−1^ (C–O stretching and O–H bending), as well as at 851 cm^−1^ (H-atoms), 651 cm^−1^, and 600 cm^−1^ (plane bending) [16]. While in the region comprising 997–706 cm^−1^ it is related to the vibratory modes of the d-glucopyranosyl ring and the skeletal modes of the pyranose ring [7]. Likewise, chitosan (CH) exhibits a series of bands at 898, 1034, and 1088 cm^−1^, corresponding to the stretching vibrations of the C–O group, and at 1153 cm^−1^, corresponding to the asymmetric stretching of C–O–C bridge, also related to the saccharide structure [17]. Typical vibration bands for amides I and II were observed at 1646 cm^−1^ for C=O stretch, 1554 cm^−1^ for flexion –NH_2_, and 1378 cm^−1^ flexion –CH_2_. The absorption band at 3409 cm^−1^ (strong and broad) was attributed to the stretching vibration of –OH, the stretching vibration of –NH, and the intermolecular hydrogen bonds of other polysaccharides. Figure 2 depicts the possible intra- and intermolecular interactions between the different components of biodegradable blends.

Regarding the starch bands not discussed in the region of 1200–900 cm^−1^, it could be inferred that this analysis is sensitive to changes in the structure at the molecular level of starch. In general, these bands exhibited broadening due to the multiple and stronger intermolecular interactions that influence the vibration of the C–O group. Differences between the 1047 cm^−1^ bands of the crystalline regions and the 1022 cm^−1^ bands of the amorphous region were observed between the complexes [18]. Likewise, the absorption intensity of the band at 995 cm^−1^ related to the double helices of the amorphous region allowed for estimation of the degrees of order (DO, R_1047/1022_) and double helices (DD, R_995/1022_) in the mixtures (Figure 1b) [19]. In general, a linear trend is observed for DO that increases proportionally with increasing chitosan content. However, the TPS50-CH50 sample reflects a discontinuity with values higher than those corresponding to this trend. The increased DO reflects stronger intermolecular interactions between TPS and low chitosan contents (~25%). Conversely, the DD values show a rational trend where the values decrease proportionally with the increase in chitosan. In particular, TPS50-CH50 shows values equal to TPS75-CH25. It is believed that double-helix destructuring was limited in that formulation, explaining how interaction competition with the plasticizer is generated in the presence of a higher content of chitosan. An increase in structural ordering was observed in the amorphous region, promoted by the rise in chitosan content. However, a lower intramolecular interaction (H-bonds) also reflected the decrease in double helices.

### 3.4. X-ray Diffraction

**Figure 3:**
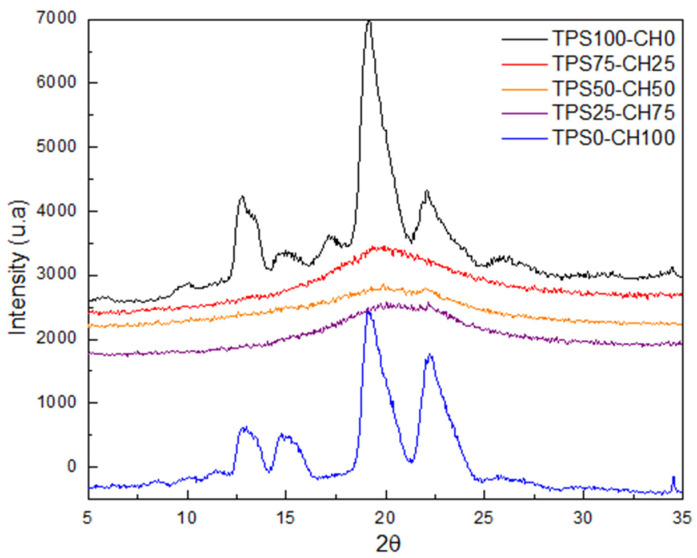
X-ray diffraction (XRD) diffractograms of starch:chitosan (TPS:CH) films.

Figure 3 presents the diffractograms of the blends of biodegradable films. In the starch film, it identifies peaks at 2θ = 12.5°, 15°, 17.5°, 20°, and 22.5°, while chitosan film shows diffraction peaks at 2θ = 13°, 15°, 20°, and 22.5°. Despite the biopolymer precursor of the blend films showing crystallinity, blends TPS75-CH25, TPS50-CH50, and TPS25-CH75 were seen to be amorphous, indicating that chitosan avoids the retrogradation of starch.

Chitosan films indicate that heat treatment evaporates the solvent of films to help reorganize the crystalline structure, as chitosan has three typical planes: 1 1 0, 0 2 0, and 1 2 0. However, chitosan films showed another peak, which it is associated with peak 1 0 1. Additionally, these peaks are shifted to lower angles, indicating an expansion of the crystal lattice of chitosan, as reported by Fan and Hu [20]. The diffractogram shows short and wide peaks with acetylated starch, which indicates ordering in the polymeric matrix. However, the peak at 2θ = 20° is characteristic of an acetylated starch, so this peak demonstrates the successful acetylation of this starch. The wide peaks corroborate disorder in the crystalline structure of starch, which could be attributed to amylose. The amylopectin double helices of starch were ruptured after the substitution of the hydroxyl groups by bulkier acetyl groups [21,22].

### 3.5. Thermal Properties

**Figure 4:**
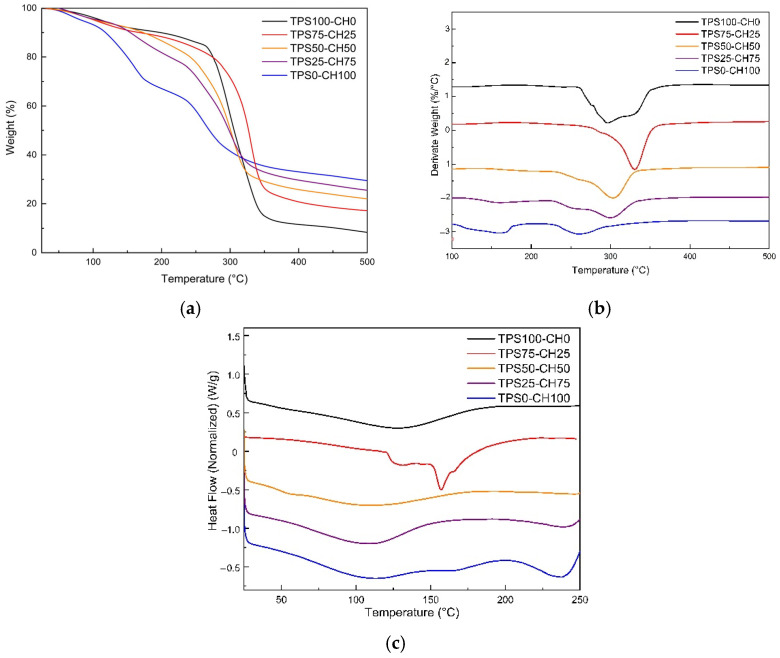
Thermal behavior of starch:chitosan films: (**a**) thermogravimetric analysis (TGA), (**b**) DTG, and (**c**) differential scanning calorimetry (DSC).

It is observed that the thermal stability (Figure 4 and Table 2) of the plasticized modified starch film (TPS100-CH0) is higher than that of the chitosan film (TPS0-CH100). There are three thermal events related to weight loss in both cases. The first is below 150 °C, where TPS100-CH00 loses 8% and TPS0-CH100 loses 20% at the same temperature. The second event occurs below 300 °C, where TPS100-CH00 and TPS0-CH100 lose 42% and 60%, respectively.

TPS50-CH50 and TPS25-CH75 blends show intermediate values to the controls. Likewise, the decomposition of chitosan into amino units (and deacetylation is complete) occurs, the glycerol evaporates (at approximately 290 °C), and the cleavage of the polymeric starch chains begins. The last event occurs beyond 300 °C, where the decomposition product residues are evident (CHOH group and starch cyclic structures arise). The best thermal performance was presented by the TPS75-CH25 blend, which manages to increase the value of the maximum degradation temperature by 11.4% for TPS100-CH0.

The thermal transitions (Figure 4) for different samples show first- and second-order transitions that relate to the gelatinization temperature for starch around 57 °C and the melting temperature of thermoplastic starch, a product of plasticization, between 110 °C and 125 °C. The glass transition temperature for chitosan is observed around 112 °C, which coincides with the reported values [23]. These researchers discussed how, in addition to plasticizer content, variability in crystallinity might be caused by factors such as water content, degree of deacetylation, hydroxyl, and available amino groups. According to the free volume theory [24], the T_g2_ of the mixtures decreases due to the plasticizer content and in the presence of low starch contents, mobilizing chitosan chains through the protonation of the amino group (H-bond). The TPS75-CH25 sample exhibits a more significant restriction in molecular mobility, with a higher and more defined Tm for TPS100-CH00. This effect is consistent with the strong interactions reported in the FT-IR analysis, as well as with the DO. Modification by the acetylation of starch causes partial molecular hydrolysis and reduces the length of glucose chains. Hence, modified starch films have a lower decomposition temperature than that of those based on native starch [3].

### 3.6. Water-Vapor Permeability (WVP)

Table 1 presents the water-vapor permeability (WVP) of the biodegradable films; starch control films (TPS100-CH0) had a value of 26.20 × 10^−11^ g·m^−1^s^−1^·Pa^−1^. The amount of water vapor permeating through the material decreased with an increase in the ratio of chitosan, the TPS25-CH75 ratio being the one that reduces it the most, with a value of 0.55 × 10^−11^ g·m^−1^s^−1^·Pa^−1^. The humidity of the material significantly modifies the barrier capacity of biodegradable materials. With higher the moisture content of the material, the barrier capacity allows for increased flow of gases. However, among the materials developed in this study, the film that absorbed the most moisture presented the best barrier to water vapor. The presence of water molecules and their interaction with the polar groups of the biopolymers through interchain hydrogen bonds progressively reduces the cohesive energy of the polymer, resulting in the plasticization of the matrix and an increase in the diffusion coefficient. Higher hydrophilicity in chitosan can explain this behavior due to the presence of –NH_3_^+^ groups, amino, and hydroxyl groups, which represent binding sites for water molecules in the chitosan chain [25]. Furthermore, the hydrophobicity of chitosan causes a reduction in interactions with water molecules, reducing the permeation of water vapor [7]. The WVP values of the films developed in this study were significantly lower than those reported for films made from different starches mixed with chitosan [16]. The materials developed in this study were a better barrier to moisture, making them a good alternative for food packaging. Results in the same order of magnitude have also been obtained for starch-lenthil flour films (16.1–18.7 × 10^−11^ g·m^−1^s^−1^·Pa^−1^) [26] and bocaiuva (*Acromonia aculeata*) flour films (0.17–0.20 × 10^−11^ g·m^−1^s^−1^·Pa^−1^) [27]. These data were compared against low-density polyethylene, which presents the highest value among conventional synthetic polymers (0.19 × 10^−11^ g·m^−1^s^−1^·Pa^−1^) [28,29,30].

### 3.7. Water-Vapor Adsorption Isotherms

**Figure 5:**
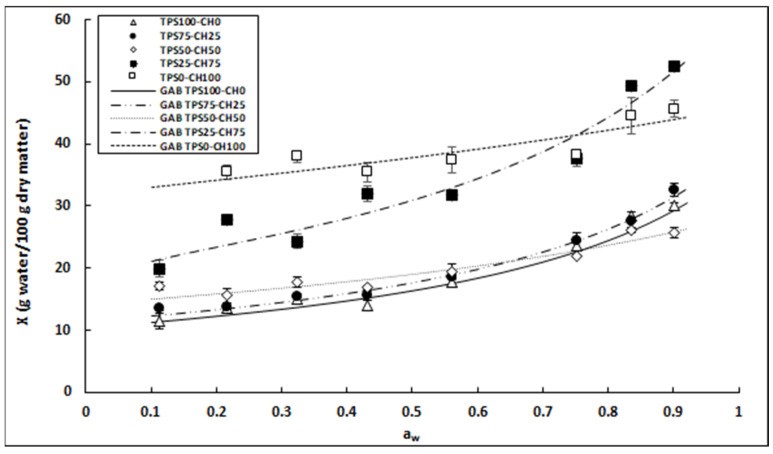
Moisture adsorption isotherms of starch:chitosan films at 25 °C, experimental data (symbols), and fitted to the GAB model (lines).

The water-vapor adsorption behavior of the materials is an important parameter that needs to be evaluated when considering use as packaging film. The moisture that these packages can adsorb when exposed to certain environmental conditions is a factor that influences and can modify their mechanical and gas-barrier properties. Figure 5 shows the experimental and fitted moisture-adsorption isotherms of TPS:CH films at 25 °C. The water-vapor adsorption of the films increased as the amount of chitosan in the formulation increased. Films of pure starch (white triangle symbol) had the lowest moisture absorption throughout the RH range, with a maximum absorption of 30% when exposed to 90% RH, whereas pure chitosan films (white box symbol) absorbed up to 46% of moisture at the same RH. TPS:CH composite films exhibited intermediate adsorption behavior compared to pure films, which occurred in environments of a***_w_*** < 0.8 (Figure 5). Above 0.8 a***_w_***, no relationship with the proportion of biopolymers was observed in the TPS25-CH75 composite films (black box symbol).

The experimental data for water-vapor adsorption were fitted to the isothermal GAB model. Table 3 depicts the GAB model parameters (*Xm*, *C*, and *k*) and the correlation coefficients (R^2^) for each biodegradable TPS:CH composite film. The parameter *Xm* corresponds to the moisture content present as the first layer of water that interacts directly with the material, called the monolayer, and is the optimal water content for the conservation and storage of dry products. An increase in the amount of water present in the monolayer of the material was observed as the chitosan ratio increased in the starch films (Table 3). According to the GAB model, the *Xm* value of the films increased as the chitosan content increased, with which it is observed that the water present in the monolayer of the material interacted significantly with this polymer. The acetylation degree has a significant impact on the first stage of adsorption of water vapor in chitosan. The number of available sites is low in chitosan, with a low degree of acetylation; thus, any form of water vapor is easily adsorbed [31]. The model obtained an adjustment value R^2^ > 0.9, indicating that this mathematical model is adequate to describe the values and the phenomenon of water-vapor adsorption observed in the evaluated materials.

Similarly, the water-adsorption behavior of biopolymers like starch may vary depending on the composition and the source of extraction [32]. Some studies have reported that the gas permeability of these materials is highly dependent on their water content [10]. High water content increases the molecular mobility of the polymer matrix, affecting the diffusion of gas molecules and increasing their permeation, contrary to the present results. In the present research, the pure starch film (TPS100-CH0) had the highest WVP (Table 1) and the least water absorption (Figure 5) compared with the pure chitosan film (TPS0-CH100) and the TPS25-CH75 composite film. Qiao and Ma [33] reported a similar result in chitosan films.

### 3.8. Mechanical Properties

**Figure 6:**
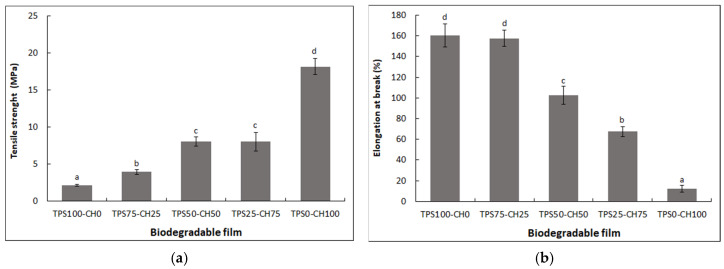
Mechanical properties of starch:chitosan biodegradable films. (**a**) Tensile strength; (**b**) elongation at break expressed as percentage. Values with a different denote significant difference (Tukey test; p < 0.05). Mean values (*n* = 3) and standard deviation.

Figure 6 presents the behavior of the mechanical properties of the TS and the percentage of elongation at break (%E) of starch–chitosan biodegradable films with three biopolymer ratios (75:25, 50:50, and 25:75) and the pure films (starch and chitosan). In the first instance, the ductility of the modified starch film (TPS100-CH0) and the stiffness of the chitosan films (TPS0-CH100) are observed. An increasing trend is observed in the TS values (Figure 6a) with increasing chitosan content. However, the TPS25-CH75 ratio remains unchanged, at 8.04 MPa, compared with the TPS50-CH50 ratio. Conversely, the %E values (Figure 6b) decrease with the incorporation of high contents of chitosan (>50%). The distribution and density of the molecular interactions that orient the functional groups of each polymer can explain the differences in the results between biopolymeric mixtures [34]. Several studies have shown that increasing chitosan concentration increases the TS of starch films [7]. This phenomenon is caused by interactions between the amino groups (NH_2_) of chitosan and OH– from starch, which promote the formation of intermolecular bonds and improve the TS behavior of materials. It has been reported that incorporation of chitosan improves the mechanical behavior of starch films when the concentration of chitosan is at least 40% [7]. The values of mechanical properties of tensile stress and elongation at break of pure polymers films (TPS100-CH0 and TPS0-CH100) are comparable to those of pure films of the same biopolymers reported by other authors [35]. When comparing TPS-CH blends with commercial biofilms, such as Ecovio P30 (TS = 34 MPa) and Mater-Bi P25 (TS = 43 MPa), a low resistance is still observed [36]. Similarly, the TS values of conventional LDPE packaging films are ~24 MPa [37]. Thus, strategies around biopolymeric mixtures or the incorporation of additives, such as reinforcements, should continue to be studied [4]. On the other hand, the elongation at break shows very satisfactory results. When compared with the same commercial samples, Ecovio P30 and Mater-Bi P25, with values of 35.4% and 10.7%, respectively, and compared to LDPE, it reaches elongations of up to 500% [38].

In general, the “rule of mixtures” is not fulfilled according to the results found in this study, despite incorporating a constant plasticizer content on the total base of the polymers. This does not exert a compatibilizing effect or homogeneous interaction with each polymer. Consequently, the interactions between glycerol and starch are stronger than those between glycerol and chitosan, as shown by the increased TS (Figure 6a) value (84.6%) of sample TPS75-CH25, maintaining ductility (elongation ~160%), compared with the starch control (TPS100-CH0). Other researchers have shown the effect of varying the proportions of glycerol on starch:chitosan = 6:4 (*w*/*w*) for mechanical properties. The higher content of plasticizer tends to increase plasticity, allowing for higher deformation; however, the TS decreases [39]. A significant trend has also been observed in the solvent-casting method because it allows scaling up to the conventional polymer processing method [40].

## 4. Conclusions

Several techniques were used to develop and characterize modified corn starch films mixed with different proportions of chitosan and plasticized with glycerol. Composite films with good workability and good characteristics were obtained to be used as food packaging material. Chitosan interacts effectively with starch, as evidenced by FT-IR, XRD, DSC, TGA, and mechanical results, which revealed a strong interaction between the two polymers. Biodegradable films based on starch–chitosan mixtures exhibited an improved water-vapor barrier compared to other starch-based materials. The TPS25-CH75 composite had the lowest permeability to this gas, although a weaker mechanical behavior than other mixtures.

The acetylation of starch was characterized using the sessile drop method, FT-IR, and XRD, and the results showed that the amylose and amylopectin double helices had been modified due to the substitution of OH groups by bulkier acetyl groups. In terms of mechanical performance, the samples showed higher rigidity as the chitosan ratio increased, except for the TPS75-CH25 blend, which showed a ductility similar to that of TPS100-CH0 but with better resistance. Furthermore, the thermal behavior of this material, in terms of the degradation temperature, was higher than that of the other formulations. These thermal transitions show miscibility between the components of the blends, with high degradation temperatures, making them susceptible and a good option for processing on a larger scale in plastic-materials production equipment.

Based on the performance of the materials obtained in this study, although their behavior is not yet on par with conventional synthetic plastics, their gas-barrier properties were found to be similar to those presented by some low-density polyethylenes. However, further research is necessary concerning the continued development of these biodegradable materials in order to bring entirely environmentally friendly materials to the market in the future with a satisfactory operation for use in the food packaging industry.

## Figures and Tables

**Table 1:** Thickness, water solubility, and water-vapor permeability of thermoplastic modified cornstarch and chitosan composite film.

Starch:Chitosan Volume Solution Ratios	Thickness (µm)	Solubility (%)	Contact Angle (°)	WVP × 10^−11^ (g·m^−1^·s^−1^·Pa^−1^)
TPS100-CH0	110.36 ± 1.61 ^d^	30.70 ± 2.35 ^a^	90.58 ± 0.40 ^d^	26.20 ± 6.43 ^e^
TPS75-CH25	162.54 ± 7.84 ^e^	47.24 ± 2.07 ^b^	86.08 ± 0.06 ^c^	4.95 ± 0.81 ^c^
TPS50-CH50	70.06 ± 0.95 ^b^	49.86 ± 5.30 ^b^	72.39 ± 0.51 ^b^	5.74 ± 0.77 ^d^
TPS25-CH75	90.46 ± 6.48 ^c^	70.92 ± 7.42 ^c^	56.79 ± 1.05 ^a^	0.55 ± 0.03 ^a^
TPS0-CH100	49.07 ± 2.59 ^a^	68.84 ± 6.68 ^c^	67.51 ± 2.02 ^b^	4.01 ± 0.52 ^b^

Values with a different letter in the same column denote significant difference (Tukey test; *p* < 0.05). Values are given as mean ± standard deviation (*n* = 10 for thickness and *n* = 3 for solubility, contact angle, and WVP).

**Table 2:** Weight losses of 10% (T_10_), weight losses of 30% (T_30_), maximum degradation (T_d1_), maximum degradation (T_d2_), glass transition (T_g_), and melting (T_m_) temperatures. Results are based on the obtained thermogravimetric analysis (TGA) and differential scanning calorimetry (DSC) thermograms for the starch-chitosan (TPS-CH) biodegradable films.

Film Sample Starch–Chitosan (TPS-CH) Ratios	T_10_	T_30_	T_d1_ *	T_d2_ *	T_g1_ *	T_m1_	T_g2_ *
	°C						
TPS100-CH0	198.3	287.7	—	295.9	55.1	127.6	
TPS75-CH25	168.0	303.0	287.3	330.7	—	131.4	156.6
TPS50-CH50	173.2	270.3	261.1	303.1	56.2	111.1 **	111.1 **
TPS25-CH75	158.3	256.0	261.0	300.0	—	108.9 **	108.9 **
TPS0-CH100	118.1	179.4	259.7	—	—		163.8

* Temperatures: subscript 1 related to chitosan and 2 related to starch. ** Widening by overlapping bands of transitions: T_m_ and T_d2_.

**Table 3:** Guggenheim–Anderson–de Boer (GAB) model parameters and regression coefficient, R^2^, calculated for biodegradable starch:chitosan films.

Starch–Chitosan Volume Solution Ratios (TPS-CH)	*X_m_*	*C*	*k*	R^2^
TPS100-CH0	10.71	1540.74	0.72	0.989
TPS75-CH25	11.35	9510.04	0.71	0.994
TPS50-CH50	14.38	15,718.83	0.49	0.975
TPS25-CH75	20.85	241.73	0.66	0.963
TPS0-CH100	32.20	4555.95	0.29	0.919

## Data Availability

The data presented in this study are available on request from the corresponding author.
